# High-fat diet induces an adaptive dysbiosis in the gut microbiota, associated with oxidative stress and the gut resistome profile in a rat model

**DOI:** 10.3389/fmicb.2026.1935516

**Published:** 2026-10-06

**Authors:** Fernanda Figueroa-Ajraz, Erik Bongcam-Rudloff, Karoll González-Pizarro, M. Alejandro Dinamarca, Luna Osses-Lobos, Ramón Sotomayor-Zárate, Claudia Ibacache-Quiroga

**Affiliations:** 1 Centro de Micro-Bioinnovación, Facultad de Farmacia, Universidad de Valparaíso, Valparaíso, Chile; 2 Escuela de Nutrición y Dietética, Facultad de Farmacia, Universidad de Valparaíso, Valparaíso, Chile; 3 Department of Animal Biosciences, Swedish University of Agricultural Sciences, Uppsala, Sweden; 4 Centro de Neurobiología y Fisiopatología Integrativa (CENFI), Instituto de Fisiología, Facultad de Ciencias, Universidad de Valparaíso, Valparaíso, Chile

**Keywords:** gut microbiota, high-fat diet, oxidative stress, rat model, resistome

## Abstract

Antibiotic resistance is one of the main public health issues worldwide; therefore, studying the factors contributing to this problem, including the reservoirs of antibiotic resistance genes, is highly relevant. Among these reservoirs, the gut microbiota stands out for its potential to disseminate antibiotic resistance genetic determinants. In this context, identifying the factors shaping the gut resistome is crucial. This study aimed to evaluate the effects of a high-fat diet (HFD) on intestinal and systemic oxidative stress in a rat model and to assess its impact on the gut microbiota, its DNA oxidative damage, and the resistome. Oxidative stress was evaluated by quantifying oxidative stress markers in the host (MDA, T-SOD, and T-GSH/GSSG) and in the gut microbiota (8-OHdG), while the impact on the gut microbiota was determined using a shotgun metagenomic sequencing approach. The effect of HFD on oxidative stress, the gut microbiota, and its resistome was evaluated after 4, 8, and 12 weeks of exposure, with and without supplementation with an antioxidant (N-acetylcysteine). Results showed that a high-fat diet generates a pro-oxidant stimulus in intestinal tissue, with increased lipid peroxidation and superoxide dismutase activity, without systemic effects. In the gut microbiota, HFD altered microbial diversity, without DNA oxidative damage. Analysis of the predominant genera revealed an increased abundance of *Mucispirillum*, *Phocaeicola*, and *Taurinivorans*, among others, and a significant reduction of *Segatella*, *Ligilactobacillus*, and *Prevotella*, which were correlated with intestinal oxidative stress. On the other hand, *Mucispirillum* showed a significant negative correlation with DNA oxidative damage in the gut microbiota. Resistome characterization showed that a high-fat diet increased the abundance of antibiotic resistance genes (ARGs) in the gut microbiota. ARGs were strongly correlated with significantly modified taxa and showed a mild correlation with oxidative stress, while specific ARGs were associated with the high-fat diet (CblA-1, the *vanH* gene in the *vanM* cluster, *vanD*, and the *vanXY* gene in the *vanE* cluster). These findings suggest an adaptive dysbiosis, characterized by a microbial community restructuring associated with oxidative stress tolerance and antibiotic resistance genes.

## 1 Introduction

Antibiotic resistance is one of the leading global public health threats, causing an estimated 1.27 million deaths directly attributable to bacterial resistance to antibiotics and contributing to 4.95 million deaths worldwide in 2019 (Murray et al., 2022). According to a recent global analysis, AMR-attributable deaths among adults aged 70 years or older increased by more than 80% between 1990 and 2021. Furthermore, global mortality attributable to AMR is projected to increase from 14.2 deaths per 100,000 population in 2022 to 20.4 deaths per 100,000 population by 2050 (Naghavi et al., 2024). Therefore, it is highly relevant to understand the potential factors contributing to it. Although antibiotic resistance has been mainly studied in pathogenic bacteria, due to their clinical/productive impact, the characterization of antibiotic resistance in environmental and commensal microbiotas has gained attention, as they play a key role as natural reservoirs of antibiotic resistance genes (Kunhikannan et al., 2021; Dabuo et al., 2025). Among these ecosystems, the gut microbiota stands out for its potential to transfer antibiotic-resistance genetic determinants to pathogenic bacteria (Göttig et al., 2015; Manoharan-Basil et al., 2022). In this scenario, identifying the factors that drive the selection and dissemination of antibiotic resistance in the gut microbiota is crucial to laying the groundwork for future prophylactic and/or compensatory strategies to counteract this global health issue.

Antibiotic resistance is a natural phenomenon identified in nature long before the introduction of these compounds to pharmacotherapy (Agudo and Reche, 2024), and the role of antibiotics in natural ecosystems is not limited to their antimicrobial effect, having a relevant function in cell communication, host-interaction, and virulence, among others (Sengupta et al., 2013). Likewise, the role of antibiotic resistance genes is not limited to survival in the presence of these molecules; they also have physiological functions such as proton exchange and environmental stress tolerance (Sengupta et al., 2013). The collection of ARGs and their precursors in pathogenic and nonpathogenic bacteria is called the resistome (Wright, 2007), and its study has attracted considerable interest in understanding the development and dissemination of antibiotic resistance within a One Health approach (Kim and Cha, 2021). Among these resistomes, the gut microbiota resistome has gained attention due to the potential capacity of this microbial community to disseminate antibiotic resistance genetic determinants (Sharma et al., 2025), therefore, understanding the stimuli that affect it is needed.

One of the main factors modulating the gut resistome is antibiotic treatment (Li et al., 2023), which exerts selective pressure on the gut microbiota. However, it is equally important to identify and address other selective pressures, such as diet, host inflammation, and exposure to biocides or heavy metals, that can also influence the abundance and dissemination of antibiotic resistance genes within the gut microbiota (Thomas et al., 2021; Stege et al., 2022; Liang et al., 2025), as well as general variables shaping the gut microbiota.

The gut microbiota is modulated by various factors, including dietary patterns, age, metabolic status, medications, and diet (Hasan and Yang, 2019). Among these, nutrition plays a pivotal role in shaping this microbial community and its function (Fan and Pedersen, 2021), with different diets affecting the gut microbiota and health (Ross et al., 2024). During the past decades, dietary patterns have drastically changed, evolving from a natural-based diet to processed foods, which are known for their high fat content, predominantly of saturated and monounsaturated fatty acids (AlOudat et al., 2021; Maldonado-Pereira et al., 2022).

Increased fat intake has been widely associated with the development of several non-communicable diseases, including type 2 diabetes mellitus and cardiovascular diseases, in which high-fat diets promote chronic inflammation (Duan et al., 2018). This dietary pattern also induces oxidative stress, both local and systemic, which also contributes to its pathogenesis (Rani et al., 2016).

Oxidative stress is defined as a disruption of redox signaling and control that occurs when the production of pro-oxidant molecules exceeds the capacity of antioxidant defense systems (Sies et al., 2017). This stress causes oxidative damage to nucleic acids (DNA and RNA), lipids, and proteins, among other molecules, altering cellular function (Sies et al., 2017). Although researchers have widely studied the impact of oxidative stress on the host (Vona et al., 2021), research on its effects on the gut microbiota has primarily focused on community structure (Semenova et al., 2024).

In bacteria, oxidative stress damages various molecules, including lipids, amino acids, DNA, and RNA (Fekrirad et al., 2026). Regarding DNA, oxidative stress generates nucleotide and backbone damage, including nucleotide oxidation and DNA breaks, which activate the SOS system, a global cellular response to counteract DNA damage, through diverse mechanisms, including low-fidelity DNA polymerases and recombination, thus promoting genetic variation (Baharoglu and Mazel, 2014).

Due to the effect of oxidative stress on genetic variation, recent studies have confirmed and expanded the role of oxidative stress as a major driver of bacterial evolvability, linking reactive oxygen species (ROS) mediated damage and SOS response induction to increased mutation rates, horizontal gene transfer, and antibiotic resistance dynamics in both experimental and gut-associated microbial systems (Rodríguez-Rojas et al., 2020; Lin et al., 2023; Qi et al., 2023). These findings support oxidative stress as a relevant selective and mutagenic force.

Genetic variation, as well as SOS induction, are linked to the development and dissemination of antibiotic resistance (Beaber et al., 2004; Dörr et al., 2009; Zabłotni et al., 2025); therefore, it is highly important to study the effects of oxidative stress induced by high-fat diets on antibiotic resistance in the gut microbiota. We hypothesize that intestinal oxidative stress from a high-fat diet promotes genetic variation and structural changes in the microbial community that ultimately promote antibiotic resistance. In this context, the present study aimed to evaluate the effects of a high-fat diet on intestinal and systemic oxidative stress in a rat model, and to assess its impact on gut microbiota composition, structure, and resistome.

## 2 Materials and methods

### 2.1 Animals and experimental design

This study used 78 adult male Sprague-Dawley rats obtained from the Animal Facility of the Faculty of Sciences, Universidad de Valparaíso, Chile, and maintained in the SAP-UVFC-1 animal facility. Animals arrived at the animal facility on postnatal day (PND) 50 and were acclimatized for 10 days before the experimental intervention began on PND 60. Rats were housed in groups of three per cage and maintained under a 12:12 h light/dark cycle, at 22 °C and 40%–70% relative humidity. *Ad libitum* food and water were provided throughout the experimental period.

The experimental design included one baseline group at pre-intervention (Pre) and four dietary treatment groups evaluated at 4, 8, and 12 weeks of intervention. The four dietary groups were control diet (CD), control diet supplemented with N-acetylcysteine (CD+NAC), high-fat diet (HFD), and high-fat diet supplemented with N-acetylcysteine (HFD+NAC). Six animals were included per group at each time point, representing independent cohorts sacrificed at each time point. The sample size for the comparison of means was estimated assuming a 95% confidence level and 80% statistical power. Additionally, a 20% increase in the estimated sample size was included to account for potential losses during the study. For the sample size calculation (n), the concentration of reactive oxygen species (ROS) in the colon under CD and HFD was used as a reference (Qiao et al., 2013). The high-fat diet consisted of Research Diets D12492 (60% kcal from fat and 20% kcal from protein, with an energy density of 5.11 kcal/g). The control diet consisted of Prolab^®^ Isopro^®^ RMH 3000 (13% fat, 23% protein, with an energy density of 3.36 kcal/g). An aqueous solution of NAC was prepared daily and administered via an oral syringe at a dose of 160 mg/kg/day.

All experimental procedures were approved by the Institutional Committee for the Care and Use of Laboratory Animals of the Universidad de Valparaíso (CICUAL-UV; approval code BEA190-23) and by the Institutional Biosafety Committee (approval code BS0323).

### 2.2 Sample collection

Unfasted rats were anesthetized with isoflurane and euthanized by decapitation according to established laboratory protocols. Blood samples were collected immediately after euthanasia and processed for serum isolation by centrifuging the sample at 1,300 rpm for 30 min at 4 °C. Serum samples were assessed immediately upon collection.

After euthanasia, the cecum was carefully excised, and cecal content was collected. Samples were immediately stored at −80 °C until DNA extraction and downstream analyses. Cecal tissue was also collected for measurements of intestinal oxidative stress after removing adherent mesenteric fat. All samples were maintained on ice during collection and stored at −80 °C for further analysis.

### 2.3 Total metagenomic DNA extraction from cecal microbiota

Total metagenomic DNA was extracted from cecal content samples using the QIAamp Fast DNA Stool Mini Kit (QIAGEN) according to the manufacturer’s instructions. DNA concentration and purity were assessed using a NanoDrop ND-1000 spectrophotometer (Thermo Fisher Scientific). DNA integrity was evaluated by electrophoresis in 1.0% agarose gel.

### 2.4 Systemic and intestinal oxidative stress

Systemic and intestinal oxidative stress were evaluated in serum and cecal tissue samples, respectively, by quantifying three oxidative stress markers: malondialdehyde (MDA), total superoxide dismutase activity (T-SOD), and the total glutathione/oxidized glutathione ratio (T-GSH/GSSG). These markers were quantified using the following commercial colorimetric kits from Elabscience: E-BC-K025-M (MDA), E-BC-K019-S (T-SOD), and E-BC-K097-S (T-GSH/GSSG), according to the manufacturer’s instructions. Total protein concentration was determined using the Bradford method (Bradford, 1976). All assays were performed in triplicate.

### 2.5 Quantification of oxidative DNA damage in cecal microbiota

Oxidative DNA damage in the cecal microbiota was assessed by quantifying 8-hydroxy-2′-deoxyguanosine (8-OHdG) in total metagenomic DNA extracted from cecal content, using the Invitrogen DNA Damage Competitive ELISA Kit (Thermo Fisher Scientific, EIADNAD), according to the manufacturer’s instructions. DNA samples were enzymatically digested according to the manufacturer’s DNA pretreatment protocol. Briefly, DNA samples were heat-denatured at 95 °C–100 °C for 10 min and cooled on ice. Samples were then digested with nuclease P1 in sodium acetate buffer containing ZnCl2 at 37 °C for 30 min. After pH adjustment with Tris-HCl, samples were treated with alkaline phosphatase at 37 °C for 30 min to obtain dephosphorylated nucleosides. Enzymes were heat-inactivated at 95 °C for 10 min, and samples were cooled on ice before ELISA analysis. All samples were analyzed in triplicate, and results were normalized to DNA amount and expressed as pg of 8-OHdG/ng DNA.

### 2.6 Metagenomic sequencing and general bioinformatic processing

Metagenomic DNA was sequenced by Macrogen, Inc., (Seoul, Republic of Korea). DNA quality assessment was performed by using a 4200 TapeStation System (Agilent Technologies). Samples with DIN > 3 and 1 μg of DNA were selected for further processing. Libraries were prepared using the TruSeq DNA PCR-Free kit (Illumina) according to the manufacturer’s protocol and sequenced using the Illumina NovaSeq X platform with a paired-end configuration (2 × 150 bp). Raw reads were initially assessed for quality using FastQC (v.0.12.1). Paired-end Illumina sequencing reads were quality-filtered and adapter-trimmed using Trimmomatic (v. 0.36) with Phred > 33 quality score encoding. Adapter contamination was removed using the TruSeq2 paired-end adapter file with the parameters ILLUMINACLIP:2:30:10, allowing up to two seed mismatches, a palindrome clip threshold of 30, and a simple clip threshold of 10. Low-quality bases at the beginning and end of reads (Phred score < 3) were trimmed, and a 4-bp sliding-window filter with a minimum average quality score of 15 was applied. Reads shorter than 36 bp after trimming were discarded. The resulting high-quality pair-end reads were used for downstream analyses.

Following quality control, host-derived sequences were removed by aligning the cleaned reads against the *Rattus norvegicus* reference genome assembly GCF_036323735.1 (GRCr8) using *Bowtie2* (v.2.3.5) with the –very-sensitive alignment mode to maximize detection of host-matching reads. The resulting alignments were generated in SAM format and subsequently converted to BAM format using SAMtools (v.1.3.1) for efficient processing.

To obtain host-depleted datasets, BAM files were filtered with SAMtools to retain only read pairs in which neither mate aligned to the host genome, and to exclude secondary and supplementary alignments. Filtered BAM files were then collated to maintain the correct read-pairing structure and reconverted into paired-end FASTQ format using the SAMtools *fastq* function, generating forward and reverse read files for each sample. Singleton reads and unpaired sequences were discarded at this stage to preserve data consistency across samples.

Finally, high-quality host-depleted reads were independently assembled per sample using MEGAHIT (v.1.2.9) with default parameters to reconstruct metagenomic contigs. The resulting assemblies were used for downstream analyses, including contig-based characterization of microbial community composition and its resistome.

Raw DNA sequences are available in the NCBI SRA repository under BioProject accession number PRJNA1493673.

All sequence statistics were generated with SeqKit2 (v. 2.13.0) (Shen et al., 2024), and are available in Supplementary Table 1. Seventy-five out of the seventy-eight samples were processed for further analyses. Two samples were discarded because no clean FASTQ files were generated (T12CD1 and T8HFD1), and one sample was discarded due to its low total contig length (T4HFD4). Finally, host-free reads were aligned against contigs using BWA (v. 0.7.19-r1273) for further analysis (Li and Durbin, 2009). Contig assembly statistics were determined using QUAST (v.5.3.0) (Gurevich et al., 2013) and SAMtools *coverage* function and are detailed in Supplementary Table 2.

### 2.7 Microbiota structure and microbial diversity analysis

Taxonomic profiling was performed using Kraken2 (v.4.5.3) (Wood et al., 2019) on quality-filtered paired-end reads. Reads were classified against the Kraken2 reference database *k2_pluspf_Sep2024*, which includes bacterial, archaeal, viral, fungal, protozoan, and host genomes to enable comprehensive metagenomic assignment. Taxonomic abundance profiles were subsequently performed using Bracken (v.2.2) (Lu et al., 2022) applied to the Kraken2 reports. Bracken was applied across all taxonomic levels (from domain to species).

Taxonomic abundance and annotation tables generated from Kraken2 outputs were processed in R to construct a unified phylogenetic dataset. Taxonomic annotation tables were merged across samples and curated to retain unique taxon identifiers. To reduce noise from low-abundance features and improve robustness of downstream analyses, taxa were filtered based on a minimum total abundance threshold of >100 reads. Only taxa exceeding this threshold were retained for subsequent analyses.

Ecological analyses were performed in R (v.4.5.3). The following R packages were used for data processing, analysis, and visualization: *phyloseq* (v.4.0.3) (McMurdie and Holmes, 2013) for microbiome data integration and analysis; *dplyr* (v.1.2.1), *tidyr* (v.1.3.2), *purr* (v.1.2.2), and *tibble* (v.3.3.1) for data manipulation; *vegan* (v.2.7-5) for ecological distance calculations and multivariate statistics; *ggplot2* (v.4.0.3) for data visualization; *ggpubr* (v.1.0.0) for statistical annotation of plots; and *ggarrange* for figure assembly. All packages were used with default settings unless otherwise specified in the main text.

Microbial community analyses were performed using the phyloseq framework. Microbial community structure was evaluated using both alpha and beta diversity approaches. Alpha diversity was assessed using Observed richness, Chao1 richness, and Shannon diversity indexes calculated from the phyloseq object. Differences in alpha diversity between experimental groups were evaluated using pairwise Wilcoxon rank-sum tests with *a priori* defined group comparisons, and statistical significance was assessed at *p*-value < 0.05.

Beta diversity was evaluated using Bray-Curtis dissimilarity calculated from relative abundance data obtained after normalization of taxa counts. Relative abundance was used to account for differences in sequencing depth among samples and to compare microbial community composition. Ordination of microbial communities was performed using both non-metric multidimensional scaling (NMDS) and principal coordinates analysis (PCoA) to visualize patterns of similarity among samples. Group differences in community composition were assessed using permutational multivariate analysis of variance (PERMANOVA) with 999 permutations. Pairwise PERMANOVA comparisons between experimental groups were also performed and resulting *p*-values were adjusted for multiple testing using the Benjamini–Hochberg false discovery rate correction. Visualization of community structure was performed using ordination plots with 95% confidence ellipses to highlight clustering patterns among experimental groups.

Community composition at different taxonomic levels was evaluated using Bracken-derived abundance estimates integrated into *phyloseq* objects constructed separately for each taxonomic level. Relative abundance at the Domain, Phylum, and Genus levels was summarized across samples and experimental groups, and mean relative abundances were calculated to describe overall community structure across conditions and time points.

### 2.8 Differential abundance analysis

Differentially abundant microbial taxa were identified using two complementary approaches: Linear Discriminant Analysis Effect Size (LEfSe) (Segata et al., 2011) and the *ANCOM-BC2* package (v.2.13.2) (Lin and Peddada, 2024), implemented in R to capture both biomarker discovery and compositional bias–corrected effect size estimation.

For compositional bias-aware differential abundance analysis, the *ANCOM-BC2* framework was applied. Analyses were performed at the domain, phylum, and genus levels after taxonomic aggregation of the *phyloseq* object containing abundance data. The model included diet as a fixed effect, with samples assigned to four experimental groups (CD, CD+NAC, HFD, and HFD+NAC). Bias-corrected log-fold changes were estimated for each taxon, and statistical significance was assessed using Benjamini–Hochberg adjusted *p*-values (*q*-values < 0.05). Structural zeros were accounted for, and global tests were enabled to assess overall compositional differences.

For LEfSe analysis, the *microbiomeMarker* package (v.1.13.2) was used. Analyses were performed on a *phyloseq* object containing relative abundance data, with the dietary condition (diet) as the main grouping variable. LEfSe first applies a non-parametric Kruskal–Wallis test to identify taxa with significant differences in abundance across groups (*p*-value < 0.05), followed by linear discriminant analysis (LDA) to estimate effect sizes. A minimum LDA score threshold of 3 was applied to define discriminative taxa. Significant features were extracted, ranked according to their LDA scores, and annotated at different taxonomic levels for biological interpretation. For this analysis, relative abundance data were used to identify taxa differentially enriched between experimental groups based on their proportional representation within the microbial community, and to minimize the potential effect of differences in sequencing depth across samples.

### 2.9 Resistome analysis

The microbial resistome was characterized from assembled contigs using the Resistance Gene Identifier (RGI) tool in combination with the Comprehensive Antibiotic Resistance Database (CARD) (Alcock et al., 2023). Contig-level sequences were screened against CARD using RGI (v.6.0.3), executed in a Docker container (quay.io/biocontainers/rgi:6.0.3). To ensure high-confidence annotation, only Perfect and Strict hits were considered for downstream analyses. All retained resistance gene annotations were mapped to their corresponding Antibiotic Resistance Ontology (ARO) identifiers. ARO-level aggregation was used to construct resistome profiles by collapsing gene-level hits into ontology-based categories, facilitating consistent comparison of antibiotic resistance composition across experimental conditions and samples.

To quantify antibiotic resistance genes (ARGs), gene coordinates of the identified ARGs from RGI annotations were extracted in BED format and mapped to contig alignments for each sample. The coverage and read depth of the identified genes were then quantified using SAMtools *view* function. RGI-derived raw counts were expressed as reads per million (RPM) per sample to account for variation in community composition and sequencing depth.

### 2.10 Analysis of mobile genetic elements

Plasmids were predicted from host-free fastq pair-end sequences using SPAdes (v.4.2.0) (Bankevich et al., 2012) with the –metaplasmid option. Plasmids were recovered from seventy-three samples, and assembly and alignment statistics are detailed in Supplementary Table 3. Candidate plasmid sequences were then analyzed with geNomad (v.1.12.0) (Camargo et al., 2024) using the end-to-end option, and contigs classified as plasmids were extracted to identify antibiotic resistance genes, as described in Section “2.9 Resistome analysis.”

For mobile genetic elements analysis, protein-coding genes were predicted using Prodigal (v.2.6.3) (Hyatt et al., 2010), and protein sequences were aligned against the mobileOG-db database (v.2.0) (Brown et al., 2022) using DIAMOND (v.2.2.5.185) (Buchfink et al., 2015). Results were filtered for ≥70% query coverage and *E*-value ≤ 1e-5. Gene coordinates of the identified mobile genetic elements were extracted in BED format and mapped to alignments of the selected plasmids against host-free reads for quantification. To ensure comparability across samples, RGI-derived raw counts were expressed as reads per million (RPM) per sample to account for variation in community composition and sequencing depth.

Additionally, to identify the origin of ARG-carrying contigs and their link to mobile genetic elements, geNomad classification was performed on these specific sequences as described above.

### 2.11 Statistical analysis

All statistical analyses were performed using R Statistical Software (v4.5.3). When corresponding, data are presented as mean ± standard deviation (SD). Normality was assessed using the Shapiro–Wilk test. For normally distributed variables, differences among groups and time were evaluated using two-way analysis of variance (ANOVA), followed by Tukey’s *post hoc* multiple comparisons test. For non-normally distributed data, the Kruskal–Wallis test was applied, followed by Dunn’s multiple comparisons test with Benjamini-Hochberg adjusting for multiple testing.

Correlation studies were performed using Spearman’s rank correlation coefficient. Statistical significance was set at *p*-value < 0.05.

Microbiome and resistome statistical analyses were conducted in R using dedicated packages for microbial ecology. Alpha diversity metrics (Observed richness, Chao1, and Shannon index) were compared using Wilcoxon rank-sum tests. Beta diversity was assessed using Bray–Curtis dissimilarity, with community structure visualized by NMDS and PCoA. Group differences in microbial composition for beta-diversity analysis were tested using PERMANOVA with 999 permutations, followed by pairwise comparisons adjusted using the Benjamini–Hochberg false discovery rate (FDR) correction. Differential abundance was evaluated with *ANCOM-BC2* (FDR-adjusted *q*-value < 0.05), accounting for compositional effects, and LEfSe (Kruskal–Wallis test with LDA score ≥ 3).

## 3 Results

### 3.1 High-fat diet promotes intestinal oxidative stress in rats without significant oxidative damage in microbiota

Increased high-fat intake is a key characteristic of the Western diet and has been linked to adverse effects via oxidative stress at both local intestinal and systemic levels (Duan et al., 2018; Rohr et al., 2020). To evaluate the effect of a high-fat diet (HFD) on systemic and intestinal oxidative stress (OS) in the host, three OS markers (MDA, T-SOD, and T-GSH/GSSG) were evaluated in cecum tissue and in serum samples from animals fed with a control diet (CD) and HFD after 4, 8, and 12 weeks. According to the results (Figure 1 and Supplementary Figure 1), HFD generated changes in the OS at the intestinal level, increasing lipid peroxidation (MDA) at four (0.0344 vs. 0.0525, *p*-value = 0.0268) and 12 weeks (0.0404 vs. 0.0535, *p*-value = 0.0019) (Figure 1A). At 12 weeks, HFD also induced T-SOD activity, but no significant differences in the T-GSH/GSSG ratio were observed between diets (Figures 1B, C).

**FIGURE 1:**
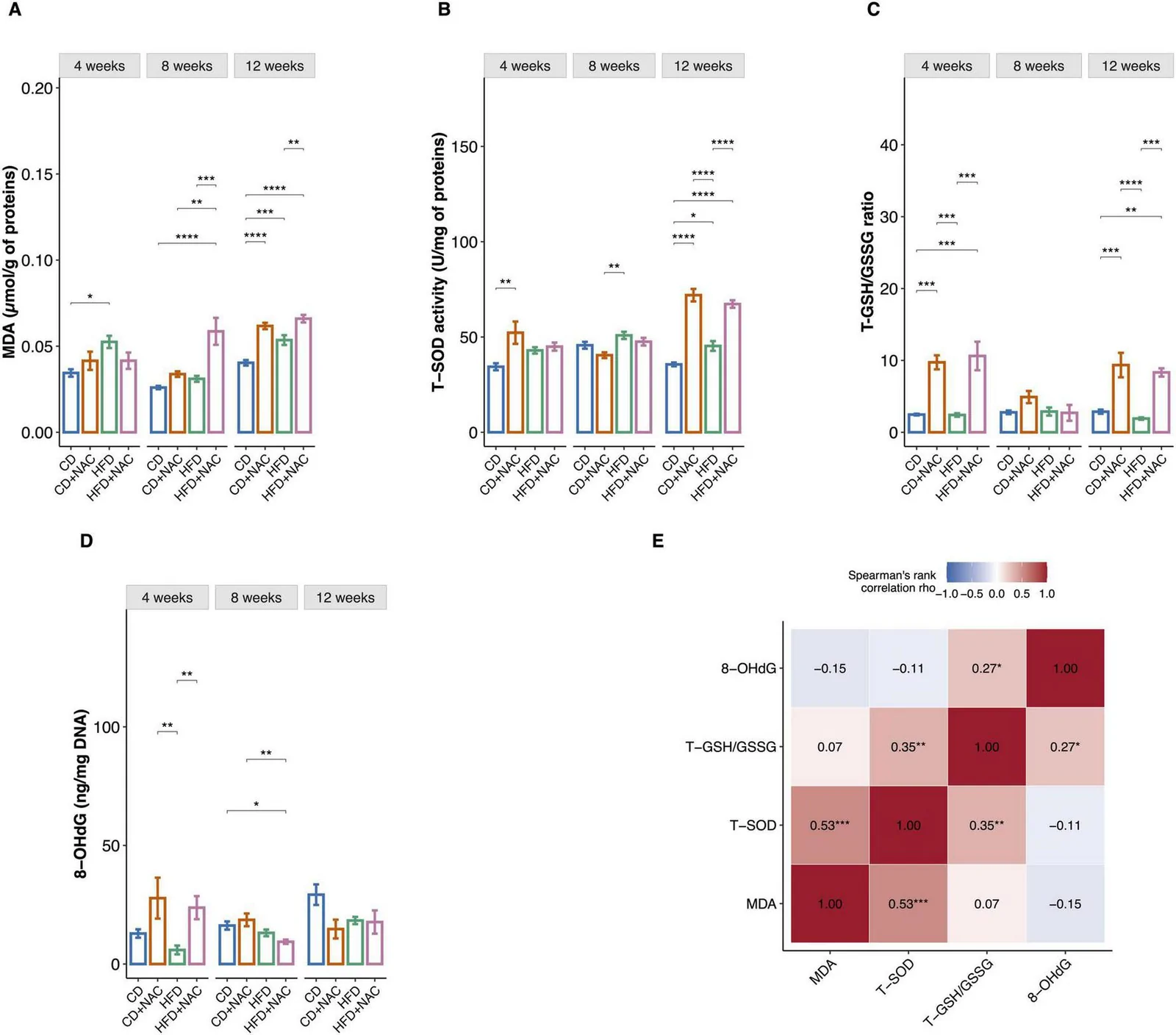
Oxidative stress markers in intestinal tissue and the gut microbiota across different exposure times. Oxidative stress markers in cecal tissue and the gut microbiota after 4, 8, and 12 weeks of treatment with a control diet (CD), a high-fat diet (HFD), and both diets supplemented with N-acetylcysteine (NAC) as an antioxidant. **(A)** Quantification of malonaldehyde (MDA) in the cecum. **(B)** Total superoxide dismutase (T-SOD) activity in the cecum. **(C)** T-GSH/GSSG ratio in the cecum. **(D)** Quantification of DNA oxidation through 8-OHdG in microbial DNA from the gut microbiota. **(E)** Correlation between oxidative stress markers and microbial DNA oxidation. Asterisks indicate statistical significance: *p-value < 0.05, **p-value < 0.01, ***p-value < 0.001, ****p-value < 0.0001.

To determine whether the impact of HFD on oxidative stress markers is reversible with antioxidant treatment, the diets were supplemented with N-acetylcysteine (NAC). This antioxidant supplementation produced significant increases in T-SOD activity and T-GSH/GSSG ratio. Additionally, this NAC treatment increased MDA concentration in the intestinal tissue of animals fed CD (0.0404 vs. 0.0617) and HFD (0.0535 vs. 0.0660).

On the other hand, at the systemic level, no significant changes in oxidative stress markers were observed between the HFD and CD groups (Supplementary Figure 1).

Intestinal oxidative stress can increase reactive oxygen species in the intestinal lumen (Qiao et al., 2013); therefore, oxidative damage in the gut microbiota was evaluated by quantifying 8-OHdG in metagenomic DNA. According to these results, no differences between high-fat and control diets were observed at any of the evaluated time points (Figure 1D).

To evaluate the association between intestinal oxidative markers and oxidative damage in microbiota, a correlation analysis was performed. As shown in Figure 1E, a moderate negative correlation was revealed between T-SOD and T-GSH/GSSG in caecum tissue (*p*-value = 0.002). Additionally, a weak positive correlation was identified between T-SOD and MDA in the caecum (*p*-value = 8.167653e-07). The correlation between intestinal OS and microbial DNA damage was also explored, showing a weak but significant positive correlation between 8-OHdG and T-GSH/GSSG ratio.

### 3.2 High-fat diet-induced changes in the gut microbiota related to oxidative stress

Diet is one of the main factors modulating the gut microbiota; therefore, the effects of a high-fat diet on the gut microbiota and microbial diversity were assessed using shotgun metagenomic sequencing. Results showed that the HFD decreased alpha diversity, as measured by the Chao1 and Observed richness indices, compared with the gut microbiota of animals fed the control diet from 4 weeks onward (Figure 2A), while the Shannon Index was unaltered. The antioxidant supplementation with NAC produced changes in alpha diversity only in the CD group (Observed and Chao1 indexes) at 12 weeks.

**FIGURE 2:**
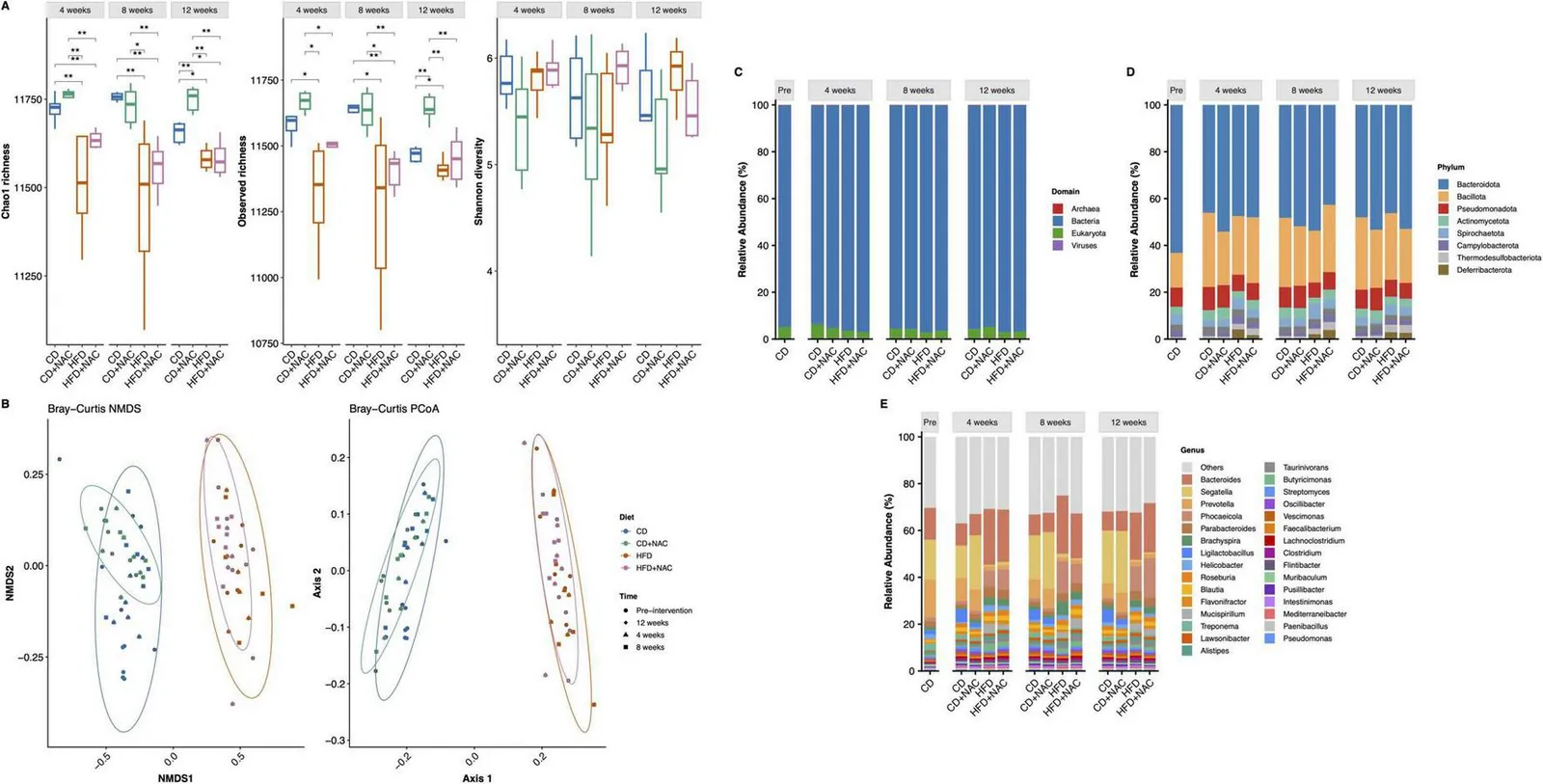
Effect of diet and antioxidant treatment on the gut microbiota. Effect of treatment on the gut microbial community after 4, 8, and 12 weeks with control diet (CD), high-fat diet (HFD), and both diets supplemented with N-acetylcysteine (NAC) as an antioxidant. **(A)** Alpha diversity measured by the Chao1, the Observed, and the Shannon indices. **(B)** Beta diversity evaluated with Bray-Curtis dissimilarity with non-metric multidimensional scaling (NMDS) and principal coordinates analysis (PCoA). **(C)** Relative abundance at the Domain level. **(D)** Relative abundance of bacterial phyla. **(E)** Relative abundance of the thirty most abundant bacterial genera in the gut microbiota. Asterisks indicate statistical significance: *p-value < 0.05, **p-value < 0.01.

In contrast, the structure of the microbial community, evaluated through beta-diversity (Bray–Curtis visualized by NMDS and PCoA) showed that the microbiota from HFD-fed rats differed significantly from those of rats receiving the CD at corresponding time points (Figure 2B). NAC supplementation had little effect on overall community structure and did not produce distinct clustering patterns, although modest time-dependent variations were observed.

The taxonomic annotation of metagenomic DNA from the gut microbiota revealed that, on average, 95.7% of the annotated sequences corresponded to Bacteria, 3.88% Eukaryota, 0.256% to Virus, and 0.191% to Archaea (Figure 2C), and no significant changes at the domain level were detected between diets at different exposure times.

At the phylum level, relative abundance of bacterial taxa showed that the main bacterial phylum in the gut microbiota was Bacteroidota (50.4%), followed by Bacillota (25.8%), Pseudomonadota (8.15%), and Actinomycetota (3.85%) and Spirochaetota (3.82%). When assessing the effect of diet on these main bacterial phyla, HFD significantly increased the relative abundance of Thermodesulfobacteriota and Deferribacterota across all evaluation times (Figure 2D). According to differential abundance analysis of bacterial phyla, expressed as logFoldChange (LFC), after 12 weeks of HFD, Deferribacterota was the phylum with the greatest increase, with an LFC of +3.43, while Thermodesulfobacteriota had an LFC of +1.41.

To characterize the gut microbiota at the genus level, the thirty most abundant genera were selected for further analyses (top taxa). Figure 2E shows the relative abundance across all experimental groups. Analysis of the differential abundance of these top taxa with respect to the control diet at 12 weeks revealed that eight out of thirty genera suffered significant changes in the HFD group (Figure 3A). Among these genera, *Ligilactobacillus* (LFC = −3.95), *Segatella* (LFC = −3.51), and *Prevotella* (LFC = −1.24) were reduced in the HFD group. In contrast, five bacterial genera were significantly increased in the HFD group compared with the control condition: *Mucispirillum* (LFC = +3.52), *Phocaeicola* (LFC = +2.08), *Taurinivorans* (LFC = +1.52), *Butyricimonas* (LFC = +1.2), and *Bacteroides* (LFC = +1.05). The antioxidant supplementation in HFD did not produce significant changes in these taxa (Supplementary Figure 2).

**FIGURE 3:**
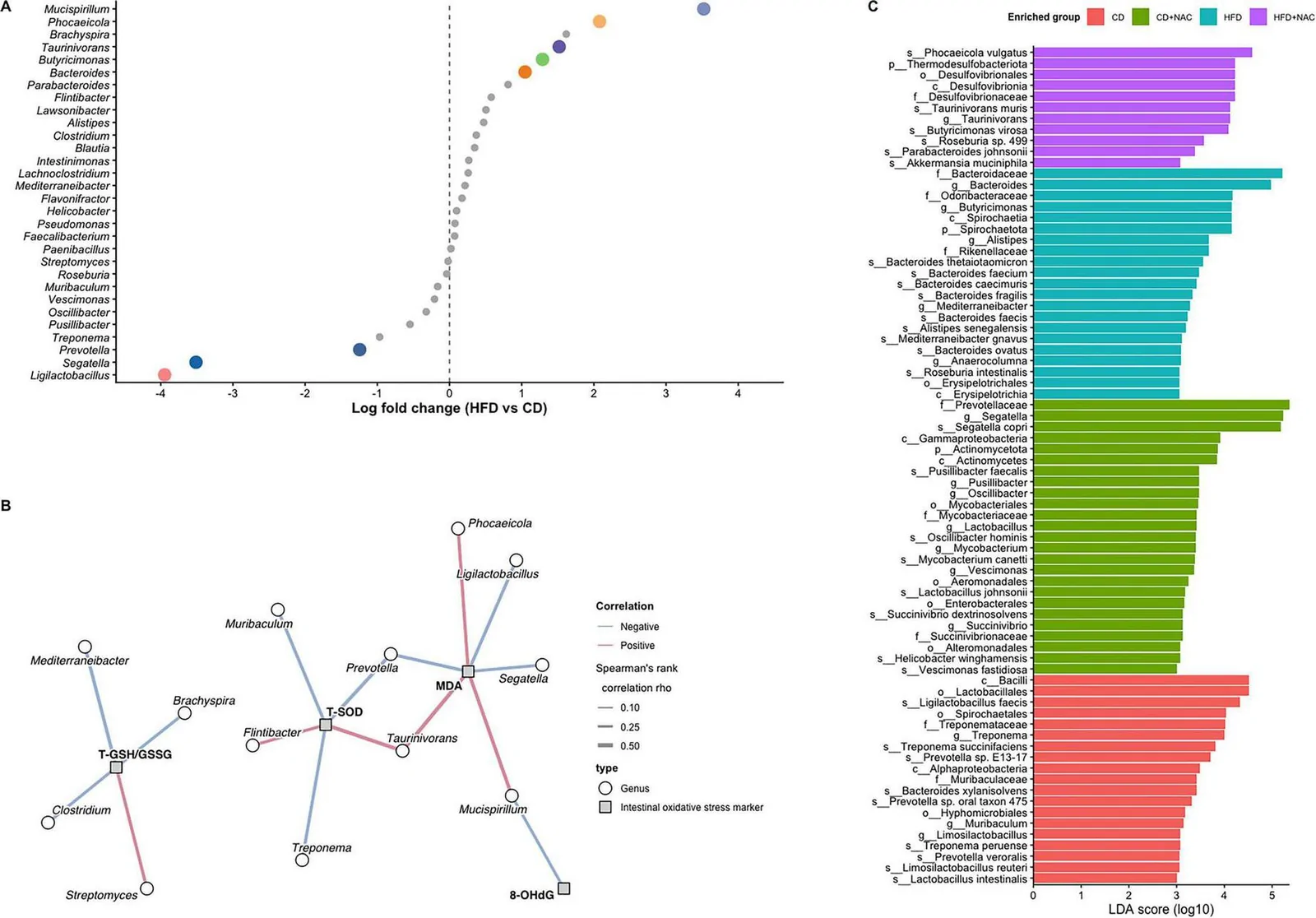
Effect of diet on differential abundance of the top taxa. **(A)** Differential abundance of top taxa in the dietary intervention groups (CD+NAC, HFD, HFD+NAC) after 12 weeks with respect to the control diet (CD) expressed as logFoldChange (LFC). Colored spheres represent statistical significance (*p*-value < 0.05). **(B)** Correlation of top taxa with intestinal oxidative stress markers. **(C)** Linear discriminant analysis effect size (LEfSe) identifying bacterial taxa that were differentially abundant among the experimental groups. Only taxa meeting the significance threshold (*p*-value < 0.05, Kruskal–Wallis test) and the selected LDA score cutoff are shown. Bar length represents the logarithmic LDA score, indicating the effect size of each taxon, while colors indicate the group in which each taxon was significantly enriched.

The gut microbiota is a microaerobic environment in which increased oxidative stress is associated with changes in microbial structure (Semenova et al., 2024). In this scenario, the potential association between intestinal oxidative stress and bacterial genera in the gut microbiota was evaluated using a correlation analysis of the identified top taxa with intestinal oxidative stress markers. As shown in Figure 3B, several slight but significant correlations were observed. T-SOD concentration in the intestinal tissue was positively correlated with *Flintibacter* and *Taurinivorans*, while *Muribaculum, Treponema, Prevotella* showed a negative one. *Prevotella* and *Taurinivorans* also had a positive and negative correlation with MDA, respectively. Additionally, this OS marker was negatively correlated with *Ligilactobacillus* and *Segatella*, and had a positive association with *Phocaeicola* and *Mucispirillum*. In contrast, this last genus was negatively correlated with DNA oxidation in the gut microbiota. As observed in Figure 3B, the T-GSH/GSSG ratio was negatively correlated with *Clostridium*, *Mediterraneibacter*, and *Brachyspira*, while *Streptomyces* had a positive association with this ratio.

In addition to the relative abundance of the top taxa, Linear Discriminant Analysis Effect Size (Lefse) was also studied, to identify potential biomarkers of each experimental group. Figure 3C presents the results of the four experimental groups with LDA > 3. According to these results, twenty-two bacterial taxa were predicted to be biomarkers of HFD. Among these, highlight the genera *Bacteroides*, *Butyricimonas*, *Mediterraneibacter*, and *Alistipes*. At the species level, most inferred biomarkers of the HFD-derived microbiota belong to the genus *Bacteroides*, including *B. thetaiotaomicron*, *B. faecium*, *B. caecimuris*, *B. fragilis*, *B. faecis*, and *B. ovatus*. In contrast, in the CD group, nineteen taxa were predicted as biomarkers, nine of which were identified at the species level. As observed in the HFD group, most inferred CD biomarkers belonged to bacterial genera among the top taxa, including *Ligilactobacillus faecis*, *Treponema succinifaciens*, *Prevotella* sp. E13-17, *Bacteroides xylanisolvens*, *Prevotella* sp. oral taxon 475, *Limosilactobacillus reuteri*, *Treponema peruense*, *P. veroralis*, and *Lactobacillus intestinalis*.

### 3.3 High-fat diet-induced changes in the gut resistome are associated with the shift in the microbial community and oxidative stress

Western diet has been previously associated with changes in the gut resistome (Keskey et al., 2025; Shen et al., 2025); therefore, the effect of HFD on this resistome was evaluated. For this purpose, antibiotic resistance genes (ARGs) were identified from gut metagenomic sequencing using the CARD database. Figure 4A shows the ARG count for each experimental group, expressed as reads per million (RPM). These results showed that a high-fat diet increased ARGs abundance in the gut microbiota from 4 weeks onward.

**FIGURE 4:**
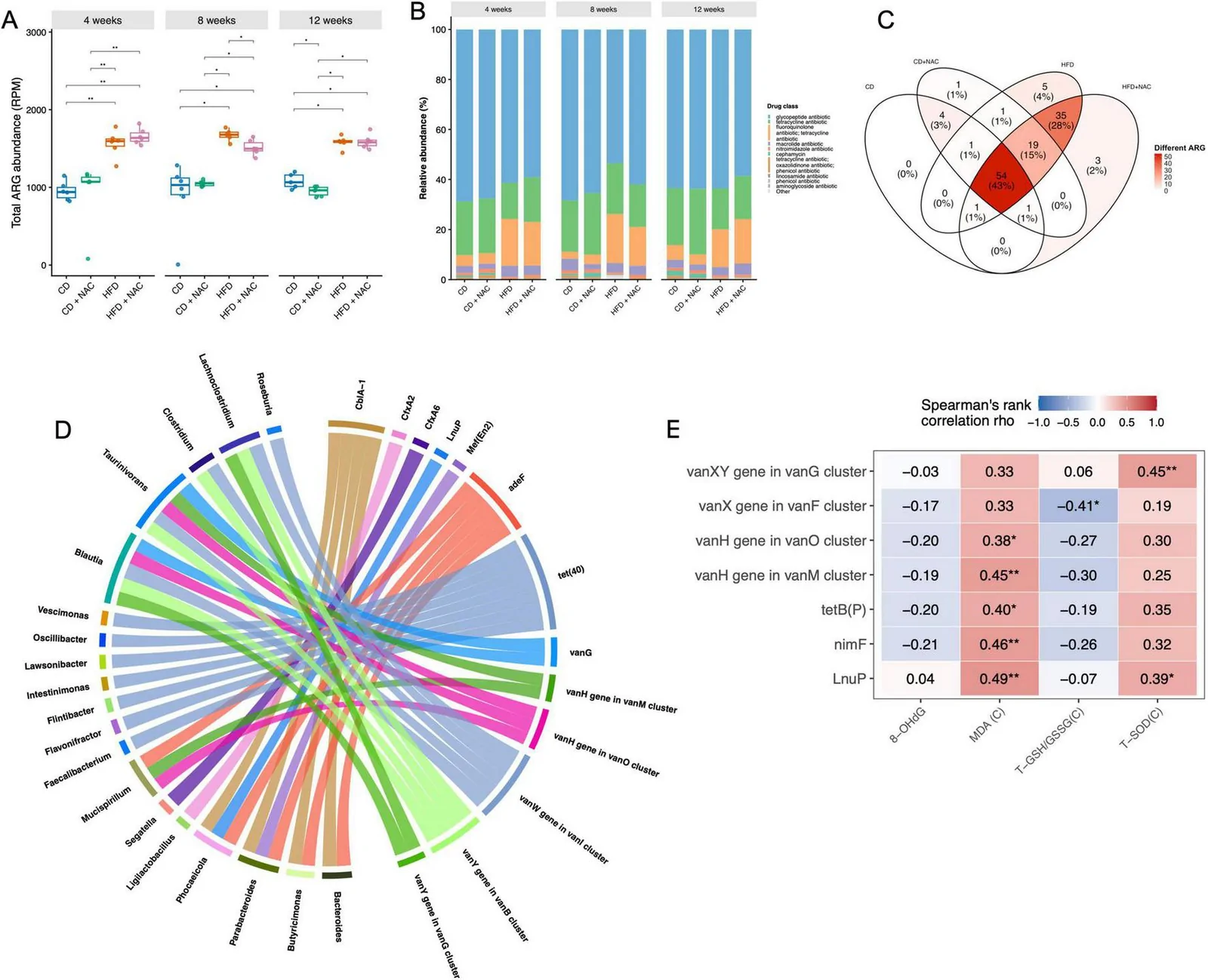
Effect of diet on gut resistome. Effect of treatment on the gut resitome after 4, 8, and 12 weeks with control diet (CD), high-fat diet (HFD), and both diets supplemented with N-acetylcysteine (NAC) as an antioxidant. **(A)** Total Antibiotic Resistance Gene (ARG) count expressed as reads per million (RPM). **(B)** Relative abundance of ARGs in the gut microbiota associated with Drug Classes. **(C)** Venn diagram of the different identified ARGs in the gut microbiota. **(D)** Strong positive correlation among top taxa and ARGs. **(E)** Significant correlation among intestinal and microbial oxidative stress and antibiotic resistance genes. Asterisks indicate statistical significance: *p-value < 0.05, **p-value < 0.01.

To characterize the rat’s gut resistome, the relative abundance of ARGs associated with drug class and antibiotic resistance mechanism was determined. These analyses revealed that most antibiotic resistance genes were associated with glycopeptide resistance (>53%), followed by tetracycline resistance genes (20%–26%) (Figure 4B). Other predominant drug classes among ARGs were fluoroquinolone antibiotic; tetracycline antibiotic, nitroimidazole, and macrolides. Regarding the resistance mechanisms, the main inferred mechanism was target alteration (>50%), followed by antibiotic target protection and efflux (Supplementary Figure 3).

Additionally, to assess whether diet and antioxidant treatment affected the resistome profile, ARGs from the CD and HFD groups, with and without NAC supplementation, were analyzed. As observed in Figure 4C, the core gut microbiome comprised fifty-four distinct antibiotic resistance genes. When comparing CD- and HFD-derived resistomes, regardless of exposure time, sixty genes were observed only in the HFD group. Four of these (CblA-1, the *vanH* gene in the *vanM* cluster, *vanD*, and the *vanXY* gene in the *vanE* cluster) were identified in at least 50% of the samples.

Supplementation with antioxidants did not alter the ARG profile in HFD-fed animals. While HFD presented 116 different ARGs, the HFD+NAC group had 113. Among these, 109 genes were common to both groups. Five ARGs were identified only in the HFD group: APH(3′)-IIa, *vanXY* gene in *vanN* cluster, *kpnH* (*Klebsiella pneumoniae*), *vanX* gene in *vanP* cluster, and *vanX* gene in *vanB* cluster, three antibiotic resistance determinants were only identified in the supplemented group: *vanXY* in the *vanC* cluster, *vanTm* in the *vanL* cluster, and *vanX* in the *vanA* cluster. Interestingly, NAC supplementation of the CD produced an increase in the number of different ARGs from sixty-three to eighty.

Because HFD and oxidative stress were linked to changes in the gut microbiota, the correlation between the top thirty bacterial genera and ARGs in the gut microbiota was explored (Supplementary Figure 3). Figure 4D shows bacterial genera that were strongly positively correlated with ARGs (Spearman’s rho > 0.7, *p*-value < 0.05). *Phocaeicola*, *Butyricimonas*, *Bacteroides*, and *Parabacteroides* were positively correlated with *adeF* and CblA. *Phocaeicola* and *Parabacteroides* were also associated with LnuP and Mef(En2), respectively. *Mucispirillum* was correlated with *adeF*, *vanH* in the *vanM* and *vanO* clusters. *Blautia* and *Mucispirillum* showed positive correlation with *van* genes. In contrast, CfxA showed a positive association with *Segatella* and *Ligilactobacillus*, two genera decreased in HFD-fed animals.

To identify whether the gut resistome was linked to intestinal or microbial oxidative stress, correlations between OS markers and ARGs were analyzed. As shown in Figure 4E, only mild correlations were identified in the resistome. Malondialdehyde (MDA) was the OS parameter that showed the largest number of correlated ARGs, including genes associated with vancomycin (*vanH*), tetracycline (*tetB*), nitroimidazole (*nimF*), and lincosamide (LnuP) resistance. The strongest correlation was identified between the MDA ratio and LnuP. Regarding microbial OS, 8-OHdG was not associated with any ARG.

### 3.4 Diet induces changes in the abundance of mobile genetic elements at early exposure times

Mobile genetic elements (MGEs) contribute to genetic variation and the dissemination of antibiotic resistance genes (Partridge et al., 2018); therefore, we determined MGEs abundance across experimental groups at different time points. Figure 5A shows that total MGEs abundance in the gut microbiota increased significantly after 4 and 8 weeks of a high-fat diet compared with the control condition. Nevertheless, after 12 weeks, no difference was detected. After 4 weeks, the five major categories were increased in HFD-fed animals (integration/excision, phage, replication/recombination/repair, stability/transfer/defense, transfer), while at 8 weeks, three of these categories were augmented (replication/recombination/repair, stability/transfer/defense, and transfer) (Figure 5B).

**FIGURE 5:**
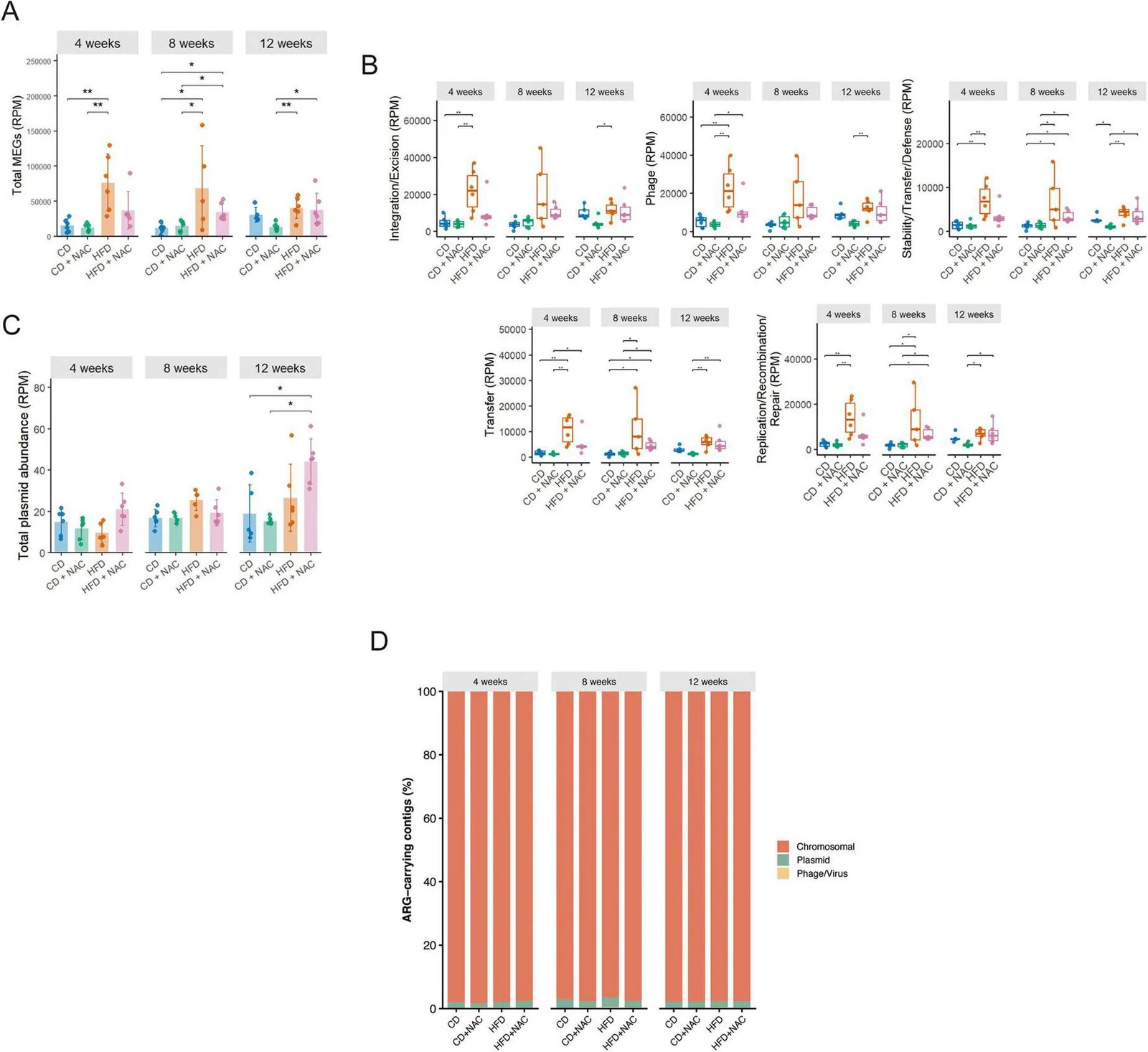
Abundance of mobile genetic elements in the gut microbiota. **(A)** Abundance of mobile genetic elements (MGEs) in the gut microbiota expressed as reads per million (RPM). **(B)** Abundance of the five major categories of MGEs in the gut microbiota, according to de mobileOG database. **(C)** Abundance of high-confidence plasmids sequences in the gut microbiota. **(D)** Proportion of chromosomal, plasmid, and phage sequences of ARG-carrying contigs from the gut microbiota. Asterisks indicate statistical significance: *p-value < 0.05, **p-value < 0.01.

To complement MGEs analysis, high-confidence predicted plasmids were quantified to determine the potential impact of diet on the abundance of these genetic elements. As shown in Figure 5C, no differences in plasmid abundance between CD and HFD were identified in this study. When analyzing antibiotic resistance genes in these sequences, we identified three antibiotic resistance genes in ten of the seventy-three samples: LnuP, TEM-116 and *tet(O)* (Supplementary Figure 4). Of these, LnuP was present in eight animals in the HFD-fed groups, with and without NAC.

Additionally, to complement the analysis of MGEs, the origin (chromosomal/plasmid/phage) of ARG-carrying contigs from the gut microbiota was analyzed. As shown in Figure 5D, more than 90% of these contigs were classified as of chromosomal origin, while less than 10% were assigned to plasmid sequences. Phage contigs represented less than 1% across samples.

## 4 Discussion

A high-fat diet is widely associated with increased oxidative stress in different tissues, contributing to the pathogenesis of several noncommunicable diseases (Liu et al., 2021; Zhao et al., 2022). In this study, HFD produced oxidative damage only at the intestinal level. Increased lipid peroxidation in the cecal tissue of HFD-fed rats was observed from the early stages (4 weeks), while significant induction of antioxidant activity (T-SOD) in response to oxidative stress was observed after 12 weeks. It is worth noting that at 8 weeks of exposure, no differences in any of the evaluated oxidative stress parameters were observed. This temporal pattern following exposure to a pro-oxidant stimulus (HFD) is consistent with the concept that oxidative stress responses are dynamic processes characterized by time-dependent activation of adaptive antioxidant mechanisms (Sthijns et al., 2016). Increased lipid peroxidation (MDA) and T-SOD activity, with an unchanged T-GSH/GSSG ratio at 12 weeks, suggest oxidative stress accompanied by a compensatory enzymatic antioxidant response without disruption of glutathione redox homeostasis. In contrast, NAC supplementation increased the T-GSH/GSSG ratio and T-SOD activity, suggesting enhanced glutathione-dependent antioxidant systems and enzymatic antioxidant defenses. However, MDA levels remained elevated, indicating persistent lipid peroxidation despite improved redox buffering capacity. This difference in NAC’s impact on oxidative stress markers is likely related to its mechanism of action. NAC’s antioxidant effect is mainly attributable to its role as a GSH precursor (Aldini et al., 2018), one of the main antioxidant mechanisms in the intestinal tissue (Circu and Aw, 2012). While the effect of this antioxidant on the T-GSH/GSSG ratio is well described, its impact on other oxidative stress markers is controversial and seems to depend on biological context, dosage, and exposure time, as reviewed by Dludla et al. (2020). Moreover, as described for several antioxidant compounds, NAC can induce oxidative stress by reducing ferric iron, depending on the biological context and dosage (Martinez et al., 2026). Although the pro-oxidant dose of NAC depends on the biological context, including the organism’s redox state (Harvey et al., 2008), the dose used in this study is within the antioxidant range, according to previous studies (De Andrade et al., 2015). In addition to lipid peroxidation by reactive oxygen species, MDA can be produced as a side product of thromboxane biosynthesis, a process upregulated under HFD in the intestine (Cai et al., 2021; Yu et al., 2026). In this scenario, MDA levels may not reflect the tissue’s redox state exclusively. Additionally, mild stress from oral NAC administration by syringe and from animal handling during dosing cannot be ruled out.

High-fat diets have been extensively linked to dysbiosis, including changes in alpha and beta diversity (Mamun et al., 2025). Our results show that this diet altered observed and estimated richness, as measured by the Observed and Chao1 indices, respectively, while Shannon diversity remained unchanged. This suggests that the high-fat diet primarily affected taxonomic richness rather than community evenness. Consistent with this, significant changes were mainly observed in low-abundance phyla, including Thermodesulfobacteriota and Deferribacteriota.

Identification of differentially abundant taxa of the core microbiota (top taxa) and potential biomarkers of dietary treatments, as well as the correlation of these genera with oxidative stress produced by the HFD, showed that *Mucispirillum* and *Phocaicola* had the highest increase in HFD-fed animals, and that these genera were significantly correlated with MDA levels in the intestinal tissue. Besides its correlation with MDA, *Mucispirillum* was negatively correlated with oxidative damage in microbial DNA. This taxon has been linked to dysbiosis in inflammatory bowel disease (Herp et al., 2019), and genomic and metatranscriptomic studies have identified antioxidant mechanisms that allow its oxidative stress tolerance (Loy et al., 2017). This antioxidant feature has also been shown to contribute to its ability to antagonize *Salmonella*’s virulence and colitis (Herp et al., 2019). On the other hand, *Phocaeicola* also shows increased tolerance to oxidative stress (Zünd et al., 2025), consistent with its selection in HFD-fed groups and correlation with oxidative stress.

The analysis of potential biomarkers from the HFD-derived microbiota highlights several *Bacteroides* species, which are increased in HFD-fed microbiota. Although this study did not show a significant correlation with oxidative stress markers, the genus *Bacteroides* has been shown to exhibit greater tolerance to oxidative stress (Zünd et al., 2025). Conversely, genera identified as potential biomarkers for CD-fed animals, such as *Prevotella*, *Segatella*, and *Ligilactobacillus*, were significantly reduced after 12 weeks of HFD, and their abundance was negatively associated with lipid peroxidation (MDA) in the intestinal tissue. These specific changes, together with the decrease in microbial diversity in the gut microbiota of HFD-fed animals, suggest that the environmental changes induced by a high-fat diet and the resulting oxidative stress in the intestinal lumen contribute to an adaptive dysbiosis, in which this microbial community undergoes a structural rearrangement in response to these selective pressures (diet and oxidative stress). The absence of microbial DNA oxidation suggests that this shift in the microbial community structure may help protect the gut microbiota against oxidative stress. Nevertheless, further studies are needed to unveil and confirm the potential protective mechanisms involved in this process.

Although changes in some of the bacterial genera identified in the top taxa have been associated with differences in nutrient availability and increased fat intake, including *Prevotella*, *Segatella*, *Ligilactobacillus*, and *Bacteroides* (Ranson et al., 2025; Yi et al., 2025), it is not possible to differentiate whether these changes are caused by direct nutrient availability, diet exposure, the physiological consequence of diet, or all. In this study, while eight of the thirty top genera showed significant changes in differential abundance, fourteen had significant correlations with oxidative stress, suggesting that shifts in the gut microbial community structure reflect a combined effect of diet and its physiological impact on the host. Furthermore, the fact that increased proportions of genera such as *Mucispirillum* and *Phocaeicola* have been observed in gut microbiota linked to other conditions characterized by increased oxidative stress, like inflammatory bowel disease (Mills et al., 2022; Ranson et al., 2025), suggests that their abundance is not exclusively regulated directly by diet (Muro et al., 2024; Marinescu et al., 2026). Interestingly, strains of *Phocaeicola* have been reported to alleviate diet-induced metabolic dysfunction-associated steatotic liver disease (MASLD) (Jin et al., 2024), and improve hyperlipidemia (Xu et al., 2023).

The fact that these oxidative stress-associated bacterial taxa were also correlated with antibiotic resistance genes identified exclusively in the resistome of HFD-fed groups, like CblA and *vanH* gene in the *vanM* cluster, proposes a link between oxidative stress, microbial structure and the resistome. CblA is a species-specific cephalosporinase from *Bacteroides* species. Despite transfer of this gene to other taxa has not been described, its abundance in the gut microbiota is of great concern (Camargo et al., 2024).

Moreover, some of these genes were also positively correlated with oxidative stress markers. Oxidative stress exerts selective pressure on microorganisms (George et al., 2019; Rodríguez-Rojas et al., 2020) and is widely associated with genetic variation and the development and dissemination of antibiotic resistance (Beaber et al., 2004; Dörr et al., 2009; Zabłotni et al., 2025), therefore, results suggest that diet, as well as oxidative stress, shape the resistome by modifying the microbial structure of the gut microbiota.

Interestingly, among the resistome of CD+NAC-fed animals, twenty-one ARGs were not identified in CD animals but were present in animals in the HFD group. This increase in ARG repertoire could be associated with environmental changes produced by NAC in the gut microbiota of CD-fed animals. As previously mentioned, NAC can exert a pro-oxidant effect depending on the biological context (Martinez et al., 2026), as evidenced by increased MDA in the CD+NAC group compared to CD. Nevertheless, the potential impact of oxidative stress and the physiological effects of NAC on ARGs in a CD context need further investigation.

While ARG abundance increased in HFD-fed animals at all evaluation time points, mobile genetic elements (MGEs) increased mainly after 4 weeks. Previous studies of these genetic elements in the gut microbiota have shown that the environment strongly modulates MGE abundance and dynamics, and that MGE diversity and abundance increase during adaptation periods, followed by a stabilization stage (Schubert et al., 2025). This agrees with our results showing increased MGEs abundance during the early stages of our experiment. Nevertheless, the contribution of these MGEs to the dissemination of antibiotic resistance genes in this study remains unknown and requires further studies.

In contrast, plasmid analysis showed no significant differences in relative abundance between CD and HFD. Furthermore, despite no differences in plasmid abundance, we identified plasmids carrying genes encoding LnuP, an ARO correlated with intestinal oxidative stress and *Phocaeicola*, a genus positively correlated with oxidative damage in the intestine. *lnu*(P) encodes a lincosamide nucleotidyltransferase and has been identified in conjugative plasmids of *Clostridium perfringens*, located in functional transposable genetic elements (Lyras et al., 2009). Interestingly, in our study, this ARO was passively linked to the genus *Phocaeicola*.

When analyzing ARG-carrying contigs to identify their origin, we found a prevalence of chromosomal sequences, consistent with direct plasmid analysis, despite its conservative approach, suggesting chromosomally encoded ARGs are the main source of these genes. However, predicting plasmids and MGEs from metagenomic data remains challenging because non-canonical genetic elements may be present; therefore, this finding should be interpreted cautiously, and biological validation is needed.

From a public health perspective, it is important to highlight that, despite metagenomic studies do not assess functionally of ARGs, the increase of ARGs and MGEs in the gut microbiota of HFD-fed animals is of great concern, considering that antibiotic resistance and obesity are two major public health issues worldwide, therefor future studies should approach the biological and clinical impact of these findings.

Altogether, these findings support the notion that HFD and its induced oxidative stress contribute to a shift in microbial community structure without direct oxidative damage at the microbial level. In this context, a more comprehensive understanding of dysbiosis is required. The operational definition in current microbiome literature relies on descriptive, high-throughput taxonomic profiling, often inferring functional collapse from taxonomic and structural divergence; however, from a microbial ecology standpoint, these concepts oversimplify the complex, non-linear dynamics of microbial communities (Hooks and O’Malley, 2017). In our rat model, the microbial structure shifts observed without oxidative damage at the gut microbiota level challenge the conventional normal-abnormal clinical narrative centered on the human host. Rather than signaling an intrinsically “damaged” state, our findings suggest that structural shifts in response to HFD and its impact on the host represent community assembly processes operating under altered habitat conditions. Within the framework of community ecology, the intestinal tract functions as a resilient yet dynamic ecosystem governed by environmental filtering and niche differentiation (Costello et al., 2012). The HFD and the derived oxidative stress act as a specific selection pressure that modifies the local microenvironment, altering parameters such as physicochemical gradients, substrate availability, or metabolic cross-feeding networks (Bäumler and Sperandio, 2016). Consequently, the observed alterations in microbial community structure reflect population-level realignments. As selection pressures shift, taxa with higher fitness under the new environmental parameters expand while others contract, reconfiguring the ecological network without compromising overall ecosystem viability (Relman, 2012).

This structural plasticity allows microbial communities to optimize or maintain genetic, metabolic, and physiological fluxes, thereby sustaining ecological resilience when facing altered environmental inputs such as an HFD (Relman, 2012). Rather than reflecting an ecosystem collapse, these dynamics represent a transition toward an alternative stable state, driven by the intrinsic adaptability of complex microbial systems. Thus, we propose the concept of adaptive dysbiosis, wherein structural reorganizations buffer the gut microbiota against direct oxidative damage, suggesting that microbial community structure may serve as a critical factor in shaping gut microbiota and, in consequence, the gut resistome.

## 5 Conclusion

The selective pressure of a high-fat diet and the resulting intestinal oxidative stress promote a shift in the gut microbiota, increasing the relative abundance of bacterial taxa associated with enhanced oxidative stress responses. This shift in the microbial community, driven by a high-fat diet and the resulting selective pressures, contributes to the abundance and profile of the gut resistome in the rat gut microbiota.

## Data Availability

The data presented in the study are deposited in the NCBI SRA repository, accession number PRJNA1493673 (https://www.ncbi.nlm.nih.gov/).
