# Supplementary material for: High-fat diet induces an adaptive dysbiosis in the gut microbiota, associated with oxidative stress and the gut resistome profile in a rat model
Source: Front Microbiol. 2026 Oct 6;17:1935516. doi: 10.3389/fmicb.2026.1935516 (PMC13680484; doi:10.3389/fmicb.2026.1935516)
Supplement: Supplementary file 1 [file Data_Sheet_1.zip › Supplementary_material/Supplementary Figure 4.pdf]

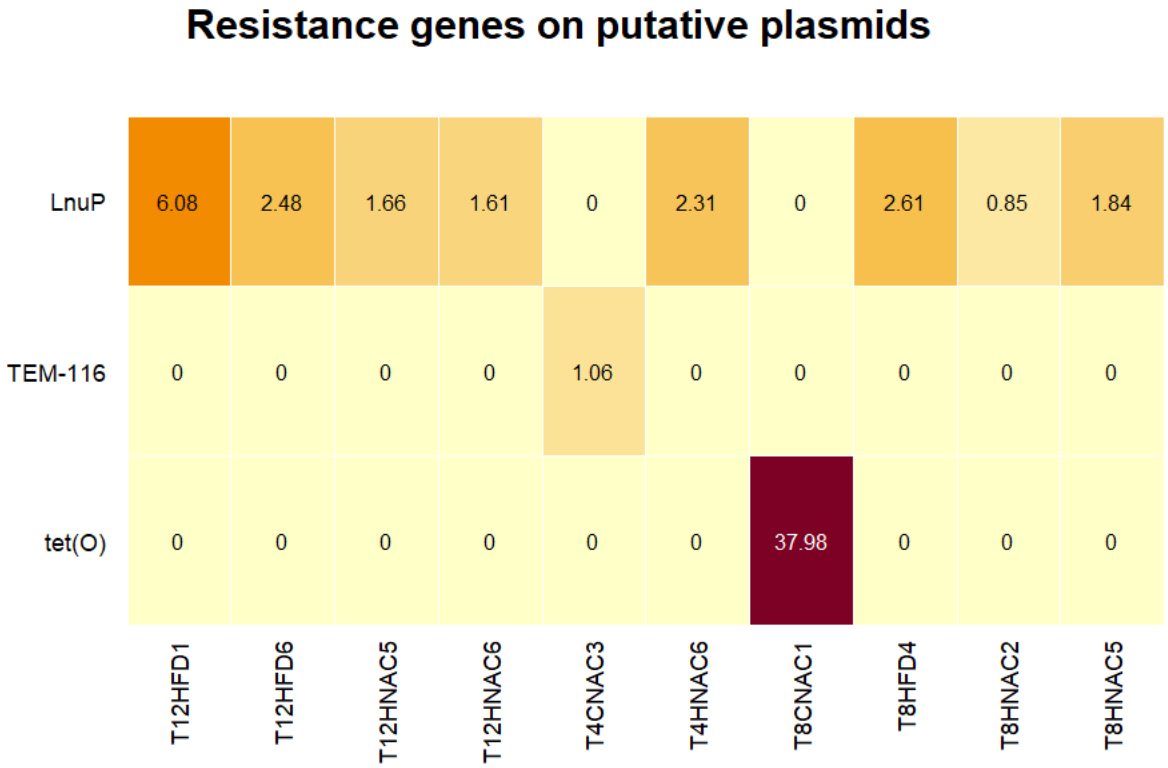

Supplementary Figure 4. Abundance of antibiotic resistance genes (ARGs) in plasmids. Abundance of ARGs in plasmids from each sample, expressed as reads per million (RPM)
