# Supplementary material for: High-fat diet induces an adaptive dysbiosis in the gut microbiota, associated with oxidative stress and the gut resistome profile in a rat model
Source: Front Microbiol. 2026 Oct 6;17:1935516. doi: 10.3389/fmicb.2026.1935516 (PMC13680484; doi:10.3389/fmicb.2026.1935516)
Supplement: Supplementary file 1 [file Data_Sheet_1.zip › Supplementary_material/Supplementary Figure 2.pdf]

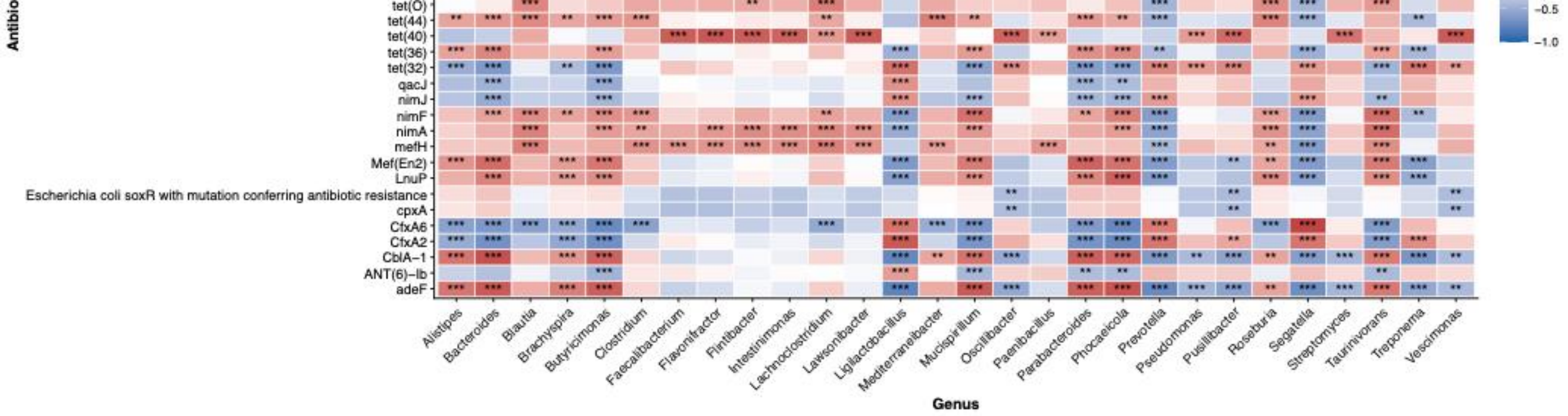

Supplementary Figure 2. Correlations between antibiotic resistance genes and the top bacterial genera. Moderate and strong correlations (Spearman`s correlation |rho| ≥ 0.4) between antibiotic resistance genes and the top 30 bacterial genera in the gut microbiota. Asterisks indicate statistical significance: **p*-value < 0.05, ** *p*-value < 0.01, *** *p*-value < 0.001.
