# Supplementary material for: High-fat diet induces an adaptive dysbiosis in the gut microbiota, associated with oxidative stress and the gut resistome profile in a rat model
Source: Front Microbiol. 2026 Oct 6;17:1935516. doi: 10.3389/fmicb.2026.1935516 (PMC13680484; doi:10.3389/fmicb.2026.1935516)
Supplement: Supplementary file 1 [file Data_Sheet_1.zip › Supplementary_material/Supplementary Figure 3.pdf]

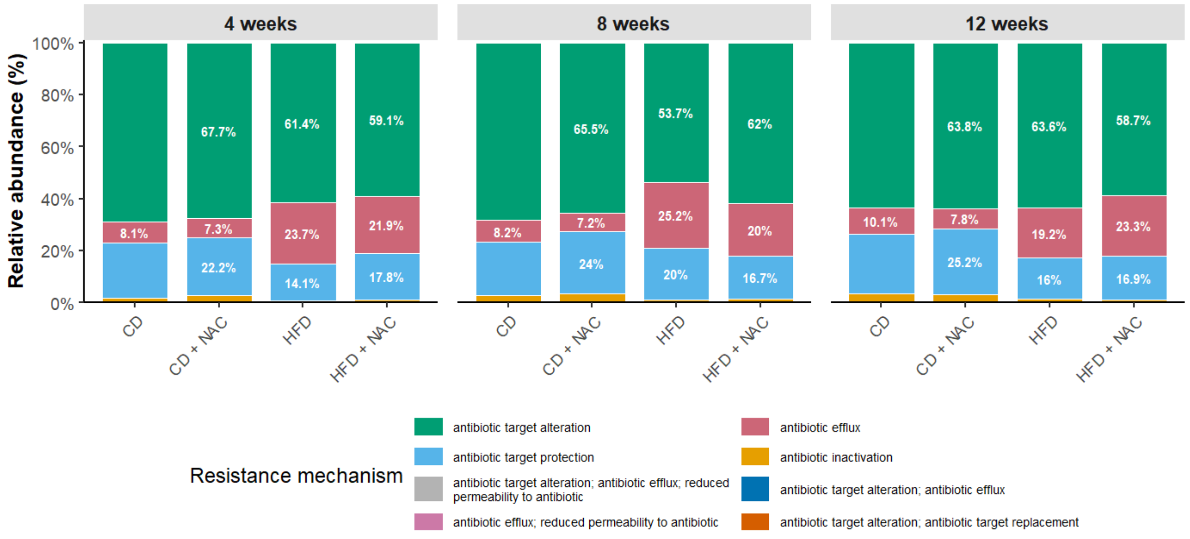

Supplementary Figure 3. Relative abundance of antibiotic resistance mechanisms in the gut microbiota. Relative abundance of antibiotic resistance mechanism associated with antibiotic resistant genes identified in gut microbiota after 4, 8, and 12 weeks of treatment with control diet (CD), high-fat diet (HFD), and both diets supplemented with N-acetylcysteine (NAC) as an antioxidant.
