# Supplementary material for: High-fat diet induces an adaptive dysbiosis in the gut microbiota, associated with oxidative stress and the gut resistome profile in a rat model
Source: Front Microbiol. 2026 Oct 6;17:1935516. doi: 10.3389/fmicb.2026.1935516 (PMC13680484; doi:10.3389/fmicb.2026.1935516)
Supplement: Supplementary file 1 [file Data_Sheet_1.zip › Supplementary_material/Supplementary Figure 1.pdf]

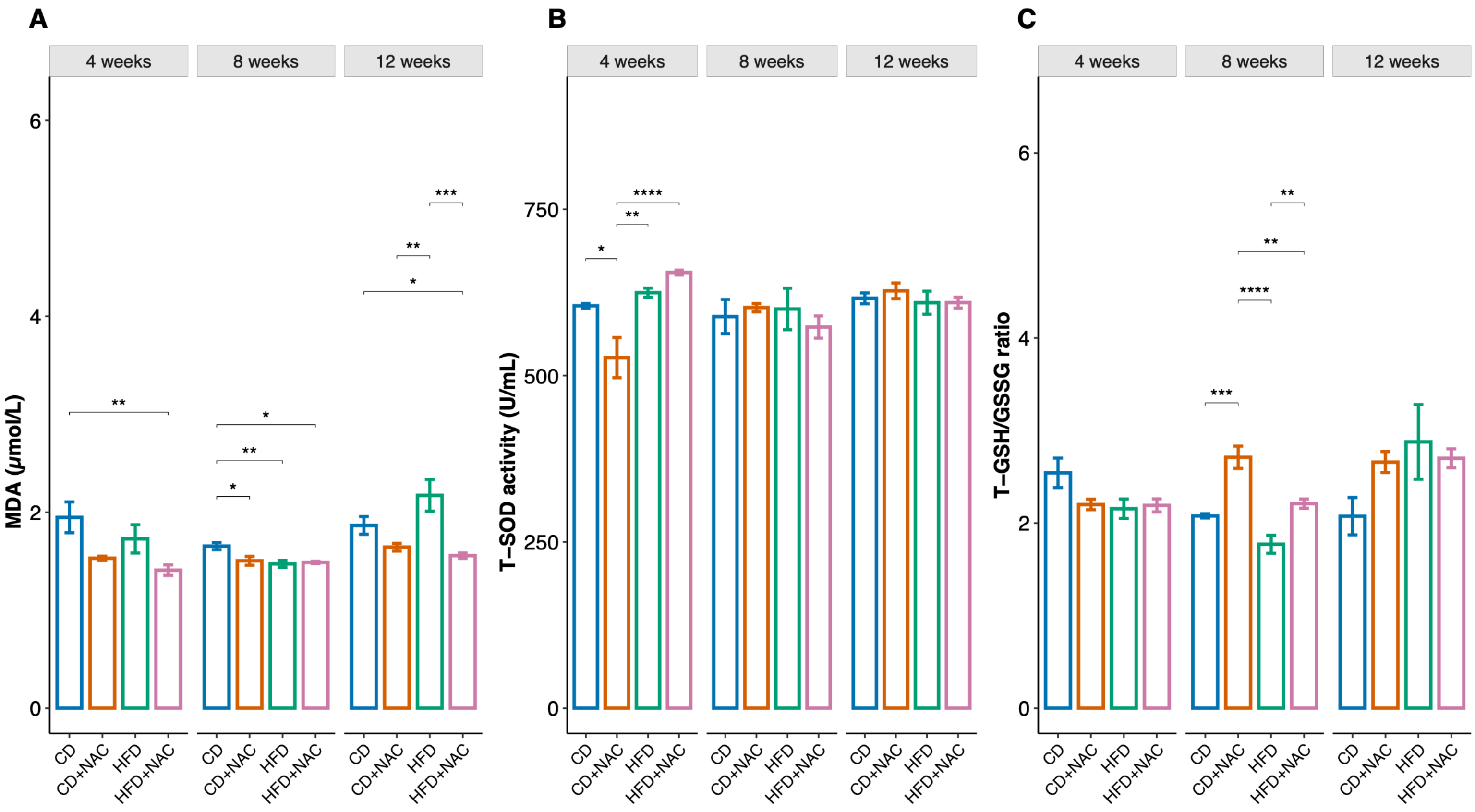

Supplementary Figure 1. Oxidative stress markers in serum across different exposure times. Oxidative stress markers in serum after 4, 8, and 12 weeks of treatment with a control diet (CD), a high-fat diet (HFD), and both diets supplemented with N-acetylcysteine (NAC) as an antioxidant. **A:** Quantification of malonaldehyde (MDA) in the cecum. **B:** Total superoxide dismutase (T-SOD) activity in the cecum. **C:** GSH/GSSG ratio in the cecum. Asterisks indicate statistical significance: *p-value < 0.05, ** p-value < 0.01, *** p-value < 0.001, and **** p-value < 0.0001.
